# The effect of COVID-19 on the presentation of thyroid disease in children

**DOI:** 10.3389/fendo.2022.1014533

**Published:** 2022-10-17

**Authors:** Rebecca McCowan, Edith Wild, Angela K. Lucas-Herald, Jane McNeilly, Avril Mason, Sze Choong Wong, S. Faisal Ahmed, M. Guftar Shaikh

**Affiliations:** ^1^ Developmental Endocrinology Research Group, University of Glasgow, Royal Hospital for Children, Glasgow, United Kingdom; ^2^ Department of Paediatric Endocrinology, Royal Hospital for Children, Glasgow, United Kingdom; ^3^ Department of Clinical Biochemistry, Queen Elizabeth University Hospital, Glasgow, United Kingdom

**Keywords:** coronavirus, virus, thyrotoxicosis, hypothyroidism, antibodies

## Abstract

**Introduction:**

Although studies suggest a potential link between COVID-19 and thyroid dysfunction in adults, there are insufficient data to confirm that association in children, and whether there is any effect on presentation to healthcare services.

**Aims:**

To identify whether presentations of thyroid dysfunction in children to a tertiary paediatric hospital changed as a result of the COVID-19 pandemic.

**Methods:**

A retrospective case note review was conducted of all children with abnormal thyroid function tests between 1^st^ January 2016 and 31^st^ December 2021 at a tertiary paediatric endocrine centre in the United Kingdom.

**Results:**

Overall, 244 children whose first presentation was within the timeframe of interest were included in this study, with a median age (range) of 11.5 (6.1, 16.8) years. Of these, 43 (18%) were hyperthyroid and 201 (82%) were hypothyroid. The greatest number of thyroid presentations occurred in 2021 (n=60, 25% of total over time period) and the fewest in 2020 (n=10, 4% of total over time period). Prior to this, the median (range) number of presentations per year was 34 (28, 39). There were no statistically significant differences in biochemistry, antibody status or other clinical characteristics between those who presented with hyperthyroidism prior to the pandemic or after. In those with hypothyroidism, baseline biochemistry was similar between the 2 groups, but the presence of other autoimmune conditions was greater pre-pandemic (17.2% vs 15.0%, p=0.03). In addition, patients were more likely to have transient thyroid dysfunction, which did not require treatment post-pandemic (70.0% vs 49.6%, p=0.0086).

**Conclusions:**

Although overall rates of presentation with thyroid dysfunction have not altered since the first wave of the COVID-19 pandemic, presentations with transient thyroid dysfunction, not requiring ongoing treatment have increased. Further research regarding the relationship between COVID-19 and thyroid function in children and young people, is needed.

## Introduction

Approximately 1-2% of children will have a thyroid disease before the age of 16 years (1). During the COVID-19 pandemic, there have been concerns regarding potential for increased rates of thyroid dysfunction, potentially due to the mechanism of entry of the virus *via* angiotensin-converting enzyme 2 (ACE2), which is highly concentrated on thyroid cells (2, 3). In addition, thyroiditis as a result of viral infection triggers pre-formed colloid release which enables raised thyroid hormone concentration, i.e. thyrotoxicosis. Both autoimmune and viral thyroiditis can destroy follicular cells, further escalating thyrotoxicosis (4). In particular, COVID-19 can cause a cytokine storm, characterised by hyperactivity of the Th1/Th17 immune response with increased production of proinflammatory cytokines, for example IL-6, which has been strongly associated with thyroiditis (5).

Studies regarding thyroid function and COVID-19 in adults suggest that the virus can contribute to increased rates and severity of thyroid dysfunction. One systematic review incorporating 1,237 adult patients, identified a positive correlation between thyroid dysfunction and clinical severity of COVID-19, with prevalence of thyroid dysfunction in patients positive for COVID-19 varying between 13-64% (6). Most commonly studies report lower Thyroid Stimulating Hormone (TSH) and free triidothyronine (fT_3_) levels (7–9) in association with the virus.

There are very limited data available to date describing the effects of the pandemic on presentation with thyroid disease in children and young people. One study from an outpatient paediatric practice in New York reported increased numbers of thyroid screening tests but with no differences in TSH in children between the ages of 6-18 years pre- and post-pandemic (10). There have also been case reports in children showing an association between COVID-19 and significant thyroid dysfunction including thyroid storm (11), as well as increased risk of admission to Paediatric Intensive Care units (12).

Of note, the COVID-19 pandemic also caused significant changes to the provision of healthcare throughout the world. For example, in one centre in the United Kingdom, nearly 1/3 of children with trisomy 21 did not receive the recommended annual TSH screening in 2020 compared to 3% in 2015 (13). A study in 25, 361 adults with thyroid disease in Japan found that thyroid control deteriorated with delayed follow up during the pandemic (14). It is possible therefore, that rates and clinical characteristics of presentation with thyroid dysfunction in all children may also differ pre- and post-pandemic.

The aim of this study was to identify whether presentations of thyroid dysfunction in children to a tertiary paediatric hospital changed as a result of the COVID-19 pandemic.

## Methods

### Patients and methods

A retrospective case note review was conducted of all children with abnormal thyroid function tests between 1^st^ January 2016 and 31^st^ December 2021 at the Royal Hospital for Children in Glasgow, Scotland. This is a tertiary paediatric hospital, which takes new patient referrals from birth to the age of 16.9 years from throughout the West of Scotland, but primarily from the Greater Glasgow and Clyde area.

Children were identified *via* a primary search of laboratory results. Inclusion criteria were: age 5.0 to 16.9 years old, TSH>5mU/L or a fT_4_>21pmol/L and recorded between 01/01/2016 to 31/12/2021. Exclusion criteria were: diagnosis of secondary thyroid disease, abnormal thyroid function result prior to 01/03/2016, or where clinical information were not available. Where a child had more than one abnormal test result recorded within the timeframe and identified in the primary search, the first abnormal result was used for analysis if it was in the study period. It was not known if the children had exposure to COVID-19, prior or current COVID‐19 infection, or COVID‐19 vaccination. However, COVID-19 vaccination was not available to children in the United Kingdom during this time period. Routinely collected hospital data were collected from the child’s electronic medical records to determine whether treatment was started for the thyroid dysfunction, as well as sex and age at the time of the blood test and the presence/absence of some key symptoms of thyroid disease. The children were seen by different members of the Paediatric Endocrinology team so absolute standards for starting treatment, for example, may differ although in all cases, this was in response to clinical condition as well as biochemistry. For the purposes of analysis, ‘pre-pandemic’ refers to results prior to 01/03/2020.

### Biochemical analyses

All biochemical analyses were performed on the Abbott Architect platform using chemiluminescent microparticle immunoassays (CMIA). The functional sensitivity for TSH was 0.01 U/L. The inter-and intra-assay coefficients of variation (CVs) were for TSH were; (inter) 3.1%, 2.0% and 2.2% and (intra) 4.1%, 2.9% and 4.7% at levels of 0.7 U/L, 5.2 U/L and 23.9 U/L. The functional sensitivity for fT_4_ was 5 pmol/L. The inter-and intra-assay CVs for FT4 were; (inter) 2.6%, 2.9% and 7.1% and (intra) 1.5%, 0.8% and 4.9% at levels of 8.9 pmol/L, 18.9 pmol/L and 33.4 pmol/L. The functional sensitivity for Total T_3_ (T_3_) was 0.4 U/L. The inter-and intra-assay CVs were for (T_3_) were; (inter) 2.6%, 1.2% and 1.3% and (intra) 4.6%, 2.2% and 1.6% at levels of 1.2 nmol/L, 2.6 nmol/L and 3.9 nmol/L. The functional sensitivities for Thyroid peroxidase (TPO) antibodies and thyroid receptor antibodies (TRAb) these assays were 1.0 U/ml and 1.3 U/l respectively The inter- and intra-assay CVs for thyroid peroxidase (TPO) antibodies were (inter) 3.2%, 3.5% and 1.8% and (intra) 5.5%, 4.6% and 4.3% at levels of 25.2 U/ml, 72.5 U/ml and 197 U/ml. The inter- and intra-assay CVs for thyroid receptor antibodies (TRABs) were; (inter) 4.8%, 1.8% and 1.1% and (intra) 5.2, 2.0 and 1.2 at levels of 2.9, 9.8 and 29.9 U/l.

### Ethics

Caldicott Guardian approval was obtained for this study.

### Statistical analyses

Data processing and statistical analysis was conducted using GraphPad Prism v 8.01. To compare differences before and after the pandemic, non-continous variables were analysed using Fisher Exact Test. Continuous variables were analysed using Mann–Whitney U test. A p-value <0.05 was regarded as statistically significant.

## Results

**Figure 1:**
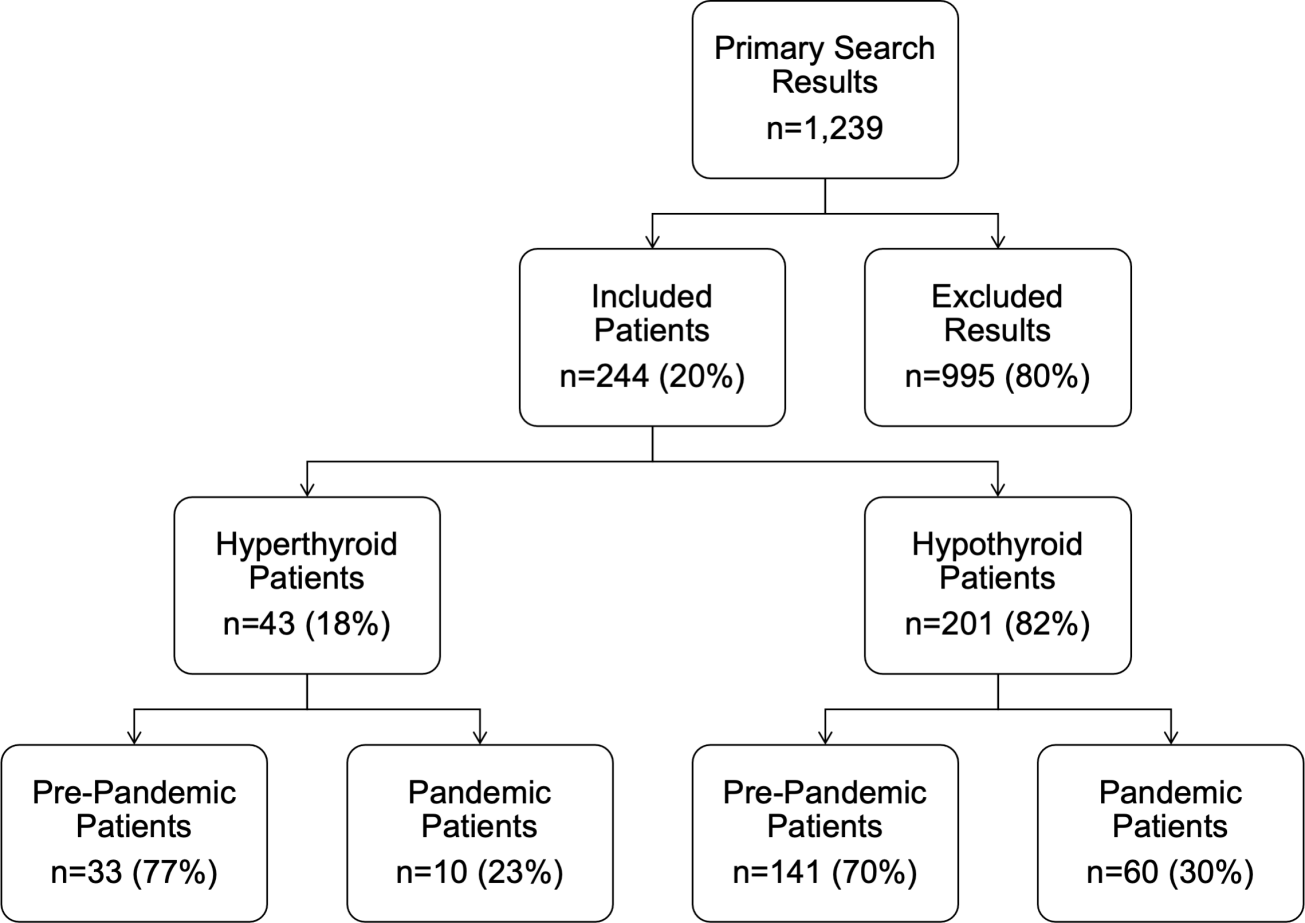
Flow diagram detailing the inclusion and exclusion of patients. ‘Pre-pandemic’ refers to the period 1^st^ January 2016 to 1^st^ March 2020. ‘Post-pandemic’ refers to the period between 2^nd^ March 2020 and 31^st^ January 2021.

The primary search identified 1,239 abnormal TSH or fT_4_ results (TSH>5mU/L or a fT4>21pmol/L) between the dates of 01/01/2016 to 31/12/2021 at the Royal Hospital for Children, Glasgow. A total of 995 (80%) of these were excluded, as they were repeat blood tests in known patients, already receiving treatment for thyroid dysfunction. Overall, 244 children whose first presentation was within the timeframe of interest were therefore included in this study, with a median age (range) of 11.5 (6.1, 16.8) years. Of these, 43 (18%) were hyperthyroid and 201 (82%) were hypothyroid (**Figure 1**). None of the children in the post-pandemic group had a history of trisomy 21, whereas 5 (3%) of the pre-pandemic group did.

**Figure 2:**
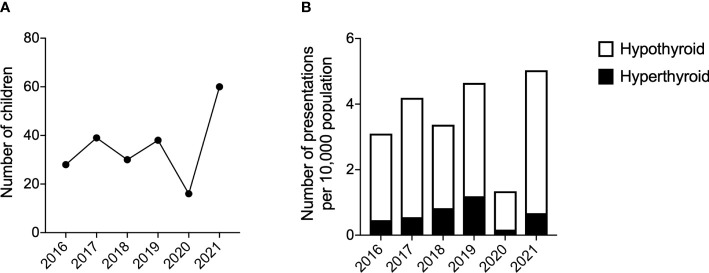
Number of presentations with thyroid dysfunction. **(A)** Number of children presenting with thyroid dysfunction per year. **(B)** Number of presentations of hyperthyroidism and hypothyroidism per 10,000 population. Years 2016-2019 used the mid-2016 population estimate for 5-15year olds in Greater Glasgow. Years 2020-2021 used the mid-2020 population estimate for 5-15year olds in Greater Glasgow. Population data from the National Records of Scotland (15).

With regards to the distribution of thyroid presentation by year within the timeframe of the study, this increased over the study period (**Figure 2**). The median (range) number of presentations with thyroid dysfunction prior to the pandemic was 34 (28, 39) per year. The greatest number of thyroid presentations occurred in 2021 (n=60, 25% of total over time period) and the fewest in 2020 (n=10, 4% of total over time period) (**Figure 2**).

### Presentation of hyperthyroidism

In those who were hyperthyroid, 33 patients presented prior to the pandemic (median (range) 8 (5-13) per year, 0.2 per week) and 10 patients presented during/after the pandemic (n=2 in 2020 and n=8 in 2021, 0.1 per week overall) (**Table 1**). There was a female preponderance in both groups. Median (range) age pre-pandemic was 12 years (7, 15) compared to 14 (7, 16) during the pandemic. There were no statistically significant differences in biochemistry or other clinical characteristics between the 2 groups. In all cases where treatment was not started, this was because repeat blood tests had normalised. There were no differences in the proportions of children presenting with unrecordably low TSH pre- and during/after the pandemic (75% vs 65%, p=0.4) or with unrecordably high fT_4_ (0% vs 0%, p>0.999).

**Table 1:** Characteristics of hyperthyroid patients.

	Pre-Pandemic n=33 (76.7%)	Pandemic n=10 (23/3%)	p-value
Female sex (%)	21 (63.6)	6 (60.0)	>0.9999
Median age (years) (range)	12 (7, 15)	14 (7, 16)	0.6729
Median TSH (mU/l) (range)	<0.01 (<0.01, 4.5)	<0.01 (<0.01, 2.38)	0.9021
Median fT4 (pmol/l) (range)	26.7 (15.7, 49.4)	34.8 (21.4, 64.4)	0.2017
Median fT3 (pmol/l) (range)	6.2 (1.4, >12.3)	7.1 (4.3, >9.2)	0.3729
Median TPO antibodies (U/ml)(range)	349.0 (1.1, >2000.0)	535.4 (<1.0, 1497.8)	0.9402
No +ve TPO antibodies (%)	21 (63.6)	7 (70.0)	>0.9999
Median TRABs (U/ml) (range)	18.0 (1.1, 77.0)	7.3 (<1.0, 33.0)	0.0800
No +ve TRABs (%)	21 (63.6)	8 (80.0)	0.4557
Goitre (%)	13 (39.4)	7 (70.0)	>0.9999
Tremor (%)	8 (24.2)	6 (60.0)	>0.9999
Menarche (% of females)	5 (23.8)	2 (20.0)	>0.9999
Autoimmune history (%)	6 (18.2)	0 (0.0)	0.1405
Family history (%)	11 (33.3)	2 (33.3)	0.1357
Treated (%)	21 (63.6)	8 (80.0)	0.457

TSH, Thyroid Stimulating Hormone; fT4, thyroxine; T3, triiodothyronine; TPO, thyroid peroxidase; TRAB, thyroid receptor antibody.

### Presentation of hypothyroidism

In the hypothyroid group 141 patients presented before the pandemic and 60 patients presented during the pandemic (0.3 per week overall) (**Table 2**). In 2021 there were 52 presentations of hypothyroidism, compared to a median (range) of 28 (13, 44) and a total of only 8 presentations during 2020. Overall, the weekly presentations of hypothyroidism increased from 0.3 new referrals per week to 0.6 post pandemic. The median (range) age in those who presented pre-pandemic was 11 years (6, 16), with the median (range) age in those presenting during/after the pandemic also being 11 (7, 16).

**Table 2:** Characteristics of hypothyroid patients (n=201) prior to the pandemic and during the pandemic.

	Pre-Pandemic n=141 (70.2%)	Pandemic n=60 (29.8%)	p-value
Female sex (%)	93 (65.9)	43 (71.7)	0.5106
Median age (years) (range)	11 (6, 16)	11 (7, 16)	0.1498
Median TSH (mU/l) (range)	8.2 (5.01, >500)	6.71 (5.01, >500)	0.8371
Median fT4 (pmol/l) (range)	11.1 (<0.01, 20.7)	11.7 (<0.01, 17.0)	0.1614
Median fT3 (pmol/l) (range)	2.0 (0.9, 4.1)	2.4 (1.6, 3.1)	0.8831
Median TPO antibodies (U/ml)(range)	373.5 (<1.0, >2000)	366.2 (<1.0, >2000)	0.7163
No +ve TPO antibodies (%)	72 (51.1)	31 (51.7)	>0.9999
Median TRABs (U/ml) (range)	1.4 (<1.0, >40.0)	<1.0 (<1.0, 7.3)	0.779
No +ve TRABs (%)	12 (8.5)	5 (8.3)	>0.9999
Goitre (%)	23 (17.2)	6 (10.0)	0.4222
Tremor (%)	0 (0)	1 (1.7)	0.3269
Menarche (% of females)	16 (23.8)	2 (20.0)	>0.9999
Autoimmune history (%)	30 (21.3)	9 (15.0)	0.0343*
Family history (%)	29 (20.6)	17 (28.3)	0.6238
Treated (%)	71 (50.4)	18 (30.0)	0.0086**

TSH, Thyroid Stimulating Hormone; fT4, thyroxine; T3, triiodothyronine; TPO, thyroid peroxidase; TRAB, thyroid receptor antibody. *p<0.05. **p<0.01. Bold mean they are statistically significant.

Presenting pre- or during the COVID-19 pandemic did not affect TSH, free T_4_ or TPO antibodies in hypothyroid patients. The presence of other autoimmune conditions was greater pre-pandemic with a prevalence of 17.2% compared to during the pandemic where there was a prevalence of 15.0%, (p=0.03). Fewer patients received treatment for thyroid dysfunction during the pandemic, with 50.4% of the patients who presented before the pandemic being treated compared to 30.0% who were commenced on treatment during/after the pandemic (p=0.0086). Again, in all cases where treatment was not started, this was because repeat blood tests had normalised. There were no differences in the proportions of children presenting with unrecordably high TSH pre- and during the pandemic (10.0% vs 11.8%, p=0.8) or with unrecordably low fT_4_ (8.5% vs 10.3%, p=0.7).

## Discussion

This retrospective observational study found that the presentation of thyroid dysfunction in children within a tertiary paediatric endocrine centre did not significantly change pre- and post-pandemic. This study adds to a small, but rapidly growing pool of evidence around the effect of the COVID-19 pandemic on thyroid function. Shidid et al. (10) recently reported no significant differences observed in percentage of abnormal TSH tests reported pre- and post- pandemic in children. The findings of our study support this statement and expand on it as we included fT_4_, fT_3_, TPO antibodies and TRABs as well as characteristics of presentation.

Within the hypothyroid cohort, more children presented with other autoimmune conditions or trisomy 21 in the pre-pandemic time frame compared to during/after the pandemic. This is contrast to studies in adults which have reported an increase in autoimmune mediated dysfunction after the pandemic (16). These data have not yet been reported in children. This may be because there was a reduction in total number of presentations of any children to healthcare services due to the constraints of the pandemic, and as such, additional autoimmune conditions, such as coeliac disease, may not have been identified during this time. The first lockdown in the UK was commenced in March 2020; therefore COVID‐19 might have been circulating in the community or the population may have changed its behaviour in response to the virus in the months before this. Indeed, overall presentations in 2020 are likely to have been lower due to limited access to healthcare during the peak of the pandemic.

In the hypothyroid cohort, there was also a reduction in the number of children requiring treatment post-pandemic. This suggests that these children may have had minor transient thyroid dysfunction, perhaps secondary to COVID-19 related thyroiditis, which then resolved prior to the need for treatment. A systematic review into thyroid dysfunction related to COVID-19 has reported thyroiditis, which spontaneously improves within 3 months of infection (17). As such, it would be interesting to determine the COVID-19 antibody status of children within this group, but this was not possible within this study. Future prospective studies should consider antibody status and its effects on health status.

This study reports data from a single health board region, which may limit its generalisability and data from paediatric endocrine centres elsewhere in the world may therefore differ accordingly. That said, the Greater Glasgow and Clyde health board reflects a geographical area with the highest density population of Scotland along with huge health discrepancies (15). Differences in the number of presentations with thyroid dysfunction may reflect challenges associated with access to health services during the pandemic.

Although overall rates of presentation with thyroid dysfunction have not altered since the first wave of the COVID-19 pandemic, presentations with transient thyroid dysfunction, not requiring ongoing treatment have increased. Further research regarding the relationship between COVID-19 and thyroid function in children and young people, is needed. Where possible, further prospective longitudinal studies should therefore be conducted.

## Data availability statement

The raw data supporting the conclusions of this article will be made available by the authors, without undue reservation.

## Author contributions

RM and EW undertook data collection and drafting of the manuscript. They contributed equally to the manuscript and should be considered as joint 1st authors. AL-H undertook data analysis and redrafting of the manuscript. JM, AM, SCW, SFA, and MGS provided data for analysis and revised the manuscript. All authors have read and approved the manuscript for submission.

## Conflict of interest

The authors declare that the research was conducted in the absence of any commercial or financial relationships that could be construed as a potential conflict of interest.

## Publisher’s note

All claims expressed in this article are solely those of the authors and do not necessarily represent those of their affiliated organizations, or those of the publisher, the editors and the reviewers. Any product that may be evaluated in this article, or claim that may be made by its manufacturer, is not guaranteed or endorsed by the publisher.
